# Editorial on Special Issue “Pharmacokinetics, Pharmacodynamics, and Drug Interactions”

**DOI:** 10.3390/pharmaceutics17020266

**Published:** 2025-02-17

**Authors:** Zhu Zhou

**Affiliations:** Department of Chemistry, York College, City University of New York, Jamaica, NY 11451, USA; zzhou1@york.cuny.edu

## 1. Introduction

Pharmacokinetics describes the absorption, distribution, metabolism, and excretion of a drug, while pharmacodynamics focuses on the effects of the drug on the body. Several intrinsic and extrinsic factors could alter a drug’s pharmacokinetics and pharmacodynamics, including other drugs/food and natural products. Pharmacokinetic drug–drug interactions (DDIs) typically examine how drug interactions impact the absorption, distribution, metabolism, and elimination of a substrate drug. Pharmacodynamic DDIs are classified into synergistic, additive, or antagonistic effects, which are determined by observing pharmacological changes [1,2]. Drug interactions can increase the drug’s exposure, reduce its therapeutic impact, or lead to unexpected side effects, which in severe cases, may be life-threatening [3]. Understanding the mechanisms underlying these interactions is critical for minimizing associated risks. A comprehensive, risk-based, and integrated approach to evaluating enzyme- and transporter-mediated DDIs has been recommended by multiple regulatory agencies including the Food and Drug Administration (FDA) and the European Medicines Agency (EMA). This approach has become a critical part of drug development and regulatory review, ensuring the safe use of new molecular entities in clinical practice [4].

## 2. Overview of the Published Articles

This Special Issue includes manuscripts that describe the results of basic, translational, and clinical research that contribute significant and novel information on pharmacokinetics, pharmacodynamics, and drug interactions.

As the use of natural products continues to rise in Western countries, research on their optimal use must advance in parallel to ensure their safe use [5]. Preclinical studies, including in vitro studies, are used to investigate the pharmacokinetics of natural products. Pedunculoside is one of the most abundant, representative, and active components in plants of the genus Ilex (Aquifoliaceae) used to treat myocardial ischemia, ameliorate hyperlipidemia, and prevent liver injury. Previous research has explored its metabolism and drug interactions, helping to evaluate and manage potential pharmacotherapy risks [6]. However, its clinical use is limited by poor bioavailability, rapid elimination, and extensive intestinal metabolism. To enhance its pharmacokinetic properties, a water-soluble inclusion complex, pedunculoside–βCDP, was developed. Comparative in vitro studies showed that both compounds were stable in simulated gastric and intestinal fluids but were metabolized by Bifidobacterium adolescentis and Bifidobacterium breve. Pharmacokinetic analysis demonstrated that pedunculoside–βCDP improved pedunculoside’s bioavailability, delayed its elimination, and reduced its intestinal metabolism (contribution 1).

In addition to in vitro studies, animal studies represent another approach for assessing the pharmacokinetics of natural products and their potential DDIs. Cannabis (*Cannabis sativa*) contains more than 500 chemicals, including more than 100 cannabinoids. The two most studied cannabinoids are the non-psychotoxic cannabidiol (CBD) and the psychoactive delta-9-tetrahydrocannabinol (THC). With the increasing use of cannabis, it is critical to understand its pharmacokinetics and DDIs. While some research has explored DDIs between other natural products or drugs and CBD/THC, more potential interactions remain to be studied. For example, kratom and CBD are commonly used to self-treat conditions like anxiety, pain, and mood disorders. Surveys suggest that there are individuals who use CBD with kratom [7]. A study in male rats examined the pharmacokinetic changes when kratom and CBD were used together. The results showed a 2.8-fold increase in the exposure of mitragynine, the primary kratom alkaloid, and elevated levels of other alkaloids. These findings indicate that CBD may increase systemic exposure to the psychoactive compounds found in kratom. Given the unknown clinical significance of these interactions, caution is advised when using both products concurrently (contribution 2).

While in vitro and animal studies provide valuable insights into DDIs, clinical trials remain the gold standard for evaluating DDIs. Acid-reducing agents are commonly used to prevent aspirin-induced gastrointestinal complications. Fexuprazan, a novel potassium-competitive acid blocker, shows potential for this purpose [8]. To assess possible pharmacokinetic and pharmacodynamic interactions, a randomized, open-label study was conducted with 22 healthy Korean participants. One group received 500 mg aspirin with 80 mg fexuprazan, while the other received fexuprazan (80 mg) alone, followed by aspirin (500 mg). No significant pharmacokinetic and pharmacodynamic interactions were observed. These results support the possible combined use of fexuprazan with aspirin (contribution 3).

Clinical DDI studies typically involve healthy young adults, while other specific populations are underrepresented. As a result, the pharmacokinetics of cannabinoids like CBD and THC in these groups remain poorly characterized, and few studies have addressed potential DDIs in such populations. This Special Issue includes a review that summarizes existing research on CBD–drug and THC–drug interactions in those populations. It outlined the mechanisms involved, discussed physiological considerations in pharmacokinetics and DDI studies, and presented modeling approaches to assess DDIs in populations such as pregnant and lactating women, pediatrics, older adults, and patients with hepatic or renal impairments (contribution 4).

This Special Issue also includes research on the impact of exogenous testosterone on the toxicokinetics and toxicodynamics of Γ-hydroxybutyric acid (GHB), which is increasingly used in the LGBTQ+ community. GHB exhibits nonlinear toxicokinetics and is a substrate for monocarboxylate transporters (MCTs). The study, conducted in ovariectomized (OVX) female and castrated (CST) male rats, evaluated the MCT1 inhibitor AR-C 155858 as a potential treatment for GHB overdose in both cisgender and transgender males. Testosterone treatment significantly altered GHB toxicokinetics, with CST rats showing lower renal clearance, higher AUC, and increased sedation, while OVX rats exhibited higher metabolic clearance. AR-C 155858 enhanced GHB clearance and improved sedative effects. This research highlights that MCT inhibition may offer a potential therapeutic strategy for GHB overdose in both cisgender and transgender males (contribution 5).

Modeling is a commonly used approach for investigating factors contributing to variability in drug pharmacokinetics. For example, population pharmacokinetic (popPK) models are commonly used to describe the time course of drug exposure in patient populations and to explore the sources of variability influencing individual drug exposure [9]. Busulfan, a key component of myeloablative conditioning regimens targeting primarily hematopoietic stem and progenitor cells, requires research on optimal exposure before hematopoietic stem cell transplantation. However, significant variability in busulfan exposure, particularly in children, has been reported. One study included in this Special Issue analyzed clinical data and busulfan plasma levels using popPK modeling from 124 pediatric patients transplanted at the University Children’s Hospital Zurich between October 2010 and February 2020. The results highlight the influence of disease and serum albumin on busulfan pharmacokinetics in pediatric patients, but substantial unexplained variability remains. Therefore, the ongoing therapeutic drug monitoring of busulfan and its subsequent dose adjustments are critical to achieving the target disease-specific exposure in pediatric patients undergoing conditioning for autologous or allogeneic hematopoietic stem cell transplantation (contribution 6).

## 3. Conclusions

In conclusion, the papers in this Special Issue highlight the importance of studying pharmacokinetics, pharmacodynamics, and DDIs to improve our understanding of the safe use of drugs and natural products. These studies, using different approaches including preclinical experiments, clinical trials, and advanced modeling techniques, are crucial for optimizing therapeutic outcomes. As the field evolves, further research incorporating methodologies such as artificial intelligence and machine learning will expand our knowledge and contribute to the development of personalized medicine.
